# Nuts and seeds – a scoping review for Nordic Nutrition Recommendations 2023

**DOI:** 10.29219/fnr.v68.10483

**Published:** 2024-02-07

**Authors:** Lars T. Fadnes, Rajiv Balakrishna

**Affiliations:** 1 Department of Global Public Health and Primary Care, University of Bergen, Bergen, Norway; 2 Bergen Addiction Research, Department of Addiction Medicine, Haukeland University Hospital, Bergen, Norway

**Keywords:** nuts, seeds, peanuts, dietary guidelines

## Abstract

**Background:**

Nuts and seeds have been part of diets in most of the world for millenniums, and they have also been consumed in the Nordic and Baltic countries for centuries. Consumption of nuts and seeds is linked with various health outcomes. Therefore, when updating the Nordic Nutrition Recommendations (NNR), summarizing the best evidence on key health outcomes from the consumption of nuts and seeds is essential.

**Objectives:**

This study aims to evaluate the updated evidence on the consumption of nuts and seeds and health outcomes regarded relevant for the Nordic and Baltic countries, as well as their dose-response relationship presented in updated systematic reviews and meta-analyses.

**Method:**

The scoping review is built on a *de novo* systematic review and an umbrella review published in 2022 on the consumption of nuts and seeds and its various health outcomes, including cardiovascular disease and diabetes.

**Results:**

Intake of nuts and seeds is associated with a lower risk of cardiovascular diseases, with evidence assessed as probable. This conclusion is mirrored by evidence from trials on biomarkers for chronic diseases. An intake of a serving of nuts of 28–30 g/day compared to not eating nuts is estimated to translate into approximately 20% relative reduction in the risks of cardiovascular disease and premature deaths. For cancers, consumption of a serving of nuts is inversely associated with cancer mortality. However, for type 2 diabetes, there are mixed and inconclusive results. Additionally, there are inverse associations between nut consumption and respiratory and infectious disease mortality. Allergies for nuts are seen among 1–2% of the population.

**Conclusion:**

Overall, the current evidence supports dietary recommendations to increase nut consumption to a serving of nuts and seeds per day for people without allergies to these foods.

## Popular scientific summary

- Nuts and seeds have a long culinary tradition, but their consumption is generally low in the Nordic and Baltic countries.
- Nuts and seeds are rich in micronutrients, unsaturated fatty acids, protein, fiber, and a range of bioactive compounds with antioxidant and antimicrobial properties.
- Current evidence indicates that the intake of nuts and seeds has a probable protective effect on cardiovascular disease.
- A daily serving of nuts is also associated with lower cancer mortality.
- The evidence is mixed and inconclusive regarding the impact of nuts and seeds and the risk of type 2 diabetes.

Tree nuts and seeds have hard shells covering them. These shells are composed of macronutrients such as fats, proteins, fibers, and minerals; micronutrients such as magnesium, selenium, and vitamin E; and a range of other active metabolites such as phenolic compounds (1–3). Peanuts have many similarities to tree nuts, but botanically they are classified as legumes (4). This review uses a culinary definition of nuts, including tree nuts, seeds, and peanuts. Nuts are highly nutrient-dense, and many of the compounds have been found to have antioxidant and antimicrobial properties (2, 5, 6). Nuts contain mostly mono- and polyunsaturated fatty acids (7). Nuts and seeds have been part of the diet in most of the world for millenniums and have also been used as traditional medicines (8). Consumption of nuts and seeds differs between settings (9, 10), with generally higher consumption seen in some African countries, Canada, parts of Europe, and the Middle East, and lower intakes in South America. Peanuts, almonds, walnuts, hazelnuts, cashews, Brazil nuts, macadamias, pistachios, sesame, and sunflower seeds are some of the frequently consumed nuts and seeds.

Nuts have been associated with a range of health outcomes including reduction in cardiovascular disease and cancers (11–15). Cardiovascular diseases and cancers are the two leading causes of death in the Nordic and Baltic countries, contributing strongly to lost life years (16, 17). On the contrary, nuts allergies and related reactions are potential unintended effects that need to be considered to achieve a positive balance between benefits and potential harms when providing guidelines to populations (18). Therefore, when updating the Nordic Nutrition Recommendations (NNR), summarizing the best evidence on health outcomes from the consumption of nuts is essential.

The objective of this scoping review is to transparently report the updated evidence on the consumption of nuts and seeds and health outcomes regarded as relevant for the Nordic and Baltic countries, as well as their dose-response relationship presented in updated systematic reviews (Box 1).

Box 1 Background papers for Nordic Nutrition Recommendations 2023

- This paper is one of many scoping reviews commissioned as part of the Nordic Nutrition Recommendations 2023 (NNR2023) project (19).
- The papers are included in the extended NNR2023 report but, for transparency, these scoping reviews are also published in *Food & Nutrition Research*.
- The scoping reviews have been peer reviewed by independent experts in the research field according to the standard procedures of the journal.
- The scoping reviews have also been subjected to public consultations (see the report to be published by the NNR2023 project).
- The NNR2023 Committee has served as the editorial board.
- While these papers are the main fundament, the NNR2023 Committee has the sole responsibility for setting dietary reference values in the NNR2023 project.

## Methods

Literature searches were screened to extract relevant evidence. No relevant, independent systematic reviews by multidisciplinary experts commissioned by national food or health authorities or international food and health organizations were identified by the NNR2023 Committee (19, 20). However, a *de novo* systematic review commissioned by the NNR2023 project was published in 2023 (21). The *de novo* systematic review screened 23,244 references from MEDLINE, Embase, Cochrane, and Scopus, including 42 papers on 28 unique cohorts with a total of 1,890,573 participants and 18 randomized controlled trials with a total of 2,266 participants. In addition to the scoping review, the authors of this scoping review and colleagues conducted an umbrella review published in 2022 in *Advances in Nutrition* (22). The umbrella review presents an overview of various health outcomes and associations with the consumption of nuts and seeds, using a culinary definition of nuts including tree nuts, peanuts, and seeds. Health outcomes of interest include cardiovascular disease, cancer, diabetes, obesity, respiratory disease, mortality, and their biomarkers for the disease. In addition, we present associations for high versus low consumption, per serving, and dose-response relationships. MEDLINE, Embase, Cochrane, and Epistemonikos were searched, and 1,546 hits were screened for systematic reviews and meta-analyses. Evidence was extracted from 89 articles on the consumption of nuts and relevant health outcomes, including 23 articles with meta-analysis on disease and mortality (Supplementary Table 1), 66 articles on biomarkers for disease, and 9 articles on allergy/adverse outcomes. Both the umbrella review and the *de novo* systematic review for NNR2023 are the main fundaments for this scoping review (22, 23). Serving sizes for nuts and seeds used in these were 28 g/day and 30 g/day, respectively.

The searches for the *de novo* systematic included articles indexed until September 20, 2021, and correspondingly until May 27, 2021, for the umbrella review. Further details of search strings, and inclusion and exclusion criteria are listed in the published articles (22, 23). The quality of systematic reviews used in the umbrella review was assessed with the AMSTAR 2 tool (19, 24) with NNR2023 adaptions, with the qualities categorized into high/moderate/low/critically low (listed as ‘A2:high’/‘A2:moderate’/‘A2:low’/‘A2:critically’). The *de novo* systematic review reviewed the strength of evidence according to the World Cancer Research Fund criteria (25).

## Diet intake in Nordic and Baltic countries

It is difficult to accurately assess the exact intake of nuts and seeds in dietary surveys because of daily variation in intake, and these food groups could be included in various foods in amounts not always known to the consumer. Almonds, hazelnuts, and walnuts have been used in Nordic countries for several centuries (26–28). In the Nordic and Baltic countries, the intake of nuts and seeds is generally relatively low (26). In Sweden, Denmark, and Estonia, the mean consumption among adults is 3–5 g/day for adults, while the estimates were 5–9 g/day for Finland and Latvia. Similarly, in Norway, the mean consumption per day is in the range of 6–7 g/day for adults and 1–2 g/day for children and adolescents (29, 30).

## Health outcomes relevant for Nordic and Baltic countries

**Fig. 1:**
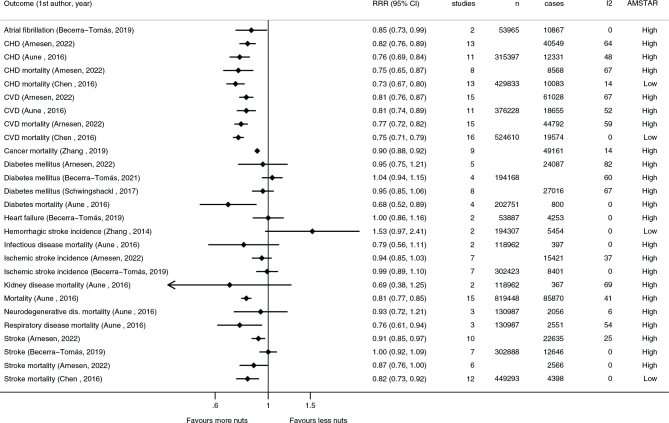
Summary of associations from the most comprehensive meta-analyses between high compared to low consumption of nuts and risk of various morbidities and mortalities. Reference is listed by first author and search year. *CHD: coronary heart disease; CVD: cardiovascular disease.

**Fig. 2:**
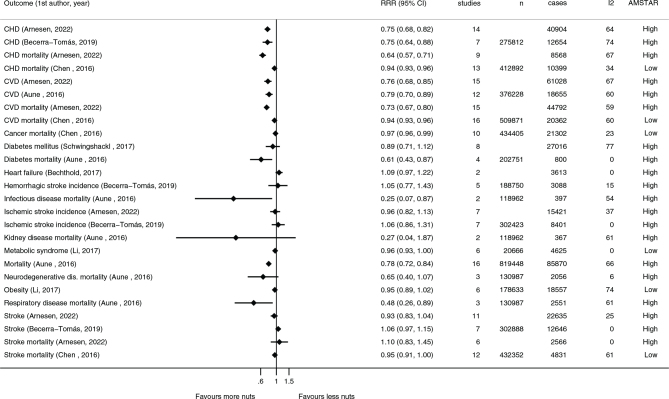
Summary of associations from the most comprehensive meta-analyses between consumption of ~30 g/day nuts and risk of various morbidities and mortalities. Reference is listed by first author and search year. *CHD: coronary heart disease; CVD: cardiovascular disease.

Cardiovascular diseases and cancers are the two leading causes of death in the Nordic and Baltic countries (16, 17). As summarized in both the *de novo* systematic review for NNR2022 and the umbrella review (21, 22), consumption of nuts and seeds is linked with a probable dose-response relationship with a reduced risk of cardiovascular disease, mostly driven by a reduction in coronary heart disease. There is also suggestive evidence for a protective effect of nut consumption on stroke and inverse associations to cancer deaths and all-cause mortality (22). There are no clear conclusions on type 2 diabetes (21, 22). The details of these and other health outcomes are presented in Figs. 1 and 2.

### Cardiovascular effects

An intake of one daily serving of nuts per day compared to not eating nuts was associated with a 19–21% relative risk (RR) reduction of cardiovascular disease (including coronary heart disease incidence and mortality, atrial fibrillation, and stroke mortality).

The RR for an intake of one daily serving of nuts compared to not eating nuts was estimated as 0.81 (95% confidence interval [CI] 0.75–0.86) for overall cardiovascular disease, 0.82 (CI 0.76–0.89) for coronary heart disease, and 0.78 (CI 0.72–0.84) for all-cause mortality (A2:high) (12, 22, 23). There was no evidence for stronger associations for nut intake beyond 30 g/day. There is suggestive evidence of nut consumption related to stroke (RR 0.91, CI 0.85–0.97; A2:high). Most studies assessing the replacement of red and processed meats with nuts have reported less cardiovascular disease when replacing meats with nuts and seeds (21).

### Cancers

The RR for cancers of all types from an intake of one daily serving of nuts compared to not eating nuts was estimated as 0.85 (CI 0.76–0.94; A2:high) (12). There is more uncertainty on the associations between nut consumption and specific cancers. Still, inverse associations have been reported between nut consumption and endometrial, colon, pancreatic, gastric, and lung cancers, with mixed results for rectal, esophageal, liver, endometrial, prostate, and breast cancer (31–34). Findings are generally similar for both tree nuts and peanuts (11, 35).

### Diabetes and body weight

Diabetes and metabolic risk factors strongly contribute to life years lost in the Nordic and Baltic countries (16, 17). For type 2 diabetes, four meta-analyses found no significant associations with nut consumption (A2: critically low, high, moderate, low) (31, 36–38), while one meta-analysis estimated a reduction in incidence with consumption of one serving of nuts per day compared to no intake (RR 0.80; CI 0.69–0.94; A2:high) (39). When adjusting for body mass index, no clear association was seen, indicating that slight weight reduction might be an effect mediator (39). As for the three first-mentioned meta-analyses, study estimates were adjusted for body mass index, and these results might have been over-adjusted. The evidence is thus inconclusive (21, 22).

### Other outcomes and study aspects

Consumption of one daily serving of nuts is associated with a reduction in mortality from respiratory disease (RR 0.48; CI 0.26–0.89) and infectious diseases (RR 0.25; CI 0.07–0.85; A2: high, evidence: low) (12). Nut consumption has further been associated with less cognitive decline and a reduced risk of depression (A2: critically, evidence: very low) (40–43).

Some trials have included nuts as a component of a complex intervention (44, 45). For several of these interventions, various components can contribute to the outcomes of interest. Further, some trials on chronic diseases might have too short follow-up to achieve the assessed health outcomes (45).

### Nut allergies and potential harms

Allergies and related adverse reactions to nuts were observed among 1–2% of adult populations, with substantial heterogeneity between studies (46). For some people, such allergies could cause severe anaphylaxis reactions that can be life-threatening if not handled promptly and properly (4, 47). Food challenge tests indicated a prevalence of allergies to tree nuts among young children of 0.03–0.2%, older children and adolescents of 0.2–2.3%, and adults of 0.4–1.4%. Allergies to peanuts and tree nuts commonly co-exist and have many similarities (4). In contrast, allergy to, for example, sesame seeds seems to be less common (48). Many tree nut reactions are milder cross-reactions to, for example, birch pollen allergy (49). Roasting generally reduces the allergenicity of some nut allergies (i.e. hazelnuts and almonds) (49). Infants at high risk of peanut allergy including children with severe eczema are recommended to introduce nuts including peanuts between 4 and 11 months, following evaluation by an appropriately trained professional (50). As avoidance of known allergens is the cornerstone to preventing allergic reactions among people with allergies, labeling food to ensure that transparency of content is essential (18).

Nuts stored in a moist environment are prone to contain fungal toxins that could be harmful (such as aflatoxin) (51). However, regulations in the processing and distribution of nuts reduce this threat.

## Mechanisms

Nuts and seeds contain many mono- and polyunsaturated fatty acids in addition to proteins and fibers (1, 52), with the former associated with a reduction in cardiovascular disease. They also contain a range of active compounds including phenolic acids, ellagitannins, phytosterols, carotenoids, and polyphenolic compounds. These compounds have antioxidant, antimicrobial, and anti-proliferative properties (5, 53, 54), which can explain some of the associations with cancers, and respiratory and infectious disease mortality. Most of these compounds are to a large degree kept through the roasting process that is common prior to consumption. However, trials assessing inflammatory markers have shown either favorable or neutral patterns (22). Some compounds such as phytates might also contribute to a reduction in the bioavailability of some nutrients in the gastrointestinal tract (51). On the contrary, nuts could also affect the microbiota. However, there is still a need for more research to understand the total impact of the consumption of nuts and seeds on microbiota (55–57).

Associations from observational studies could be linked with bias and confounders. Even though most observational studies on the intake of nuts and its health outcomes are adjusted for other risk factors such as smoking, alcohol, and activity, some residual confounding might still be involved. However, the evidence is strengthened by meta-analyses of randomized trials on the consumption of nuts and seeds, and biomarkers for disease generally mirroring findings from meta-analyses from observational studies on cardiovascular disease and cancers (Table 1) (22). This includes favorable or neutral effects of nut consumption on blood lipids, including low-density lipoprotein, endothelial function, body composition and weight, hunger and fullness, glucose and insulin, and inflammation (6, 10, 58–65). This could largely explain the inverse associations between the consumption of nuts and seeds and health outcomes such as cardiovascular and neurodegenerative diseases. Such effects were also seen in trials where red meat was replaced by nuts and legumes (66). Trials assessing nut consumption and blood pressure have either reported non-significant or slightly favorable effects on blood pressure. Still, the durations of the trials in many of these have been too short to identify potential effects (58, 67–72). Prospective cohort studies have observed inverse associations between nut consumption and hypertension (37, 38, 73).

**Table 1:** List of biomarkers for various diseases and intermediate mechanisms for various morbidities from systematic reviews and meta-analyses including cardiovascular, diabetes and weight, and other outcomes

Biomarker	Favorable	Neutral	Unfavorable
**Blood lipids**			
High-density lipoprotein	(69)	(60, 64, 67, 70, 74–88)	(65)
Low-density lipoprotein	(23, 60, 64, 65, 67, 76, 79, 81, 82, 84, 86, 88)	(69, 70, 77, 78, 80, 83, 85, 87)	
Triglycerides	(64, 67, 74–79, 81, 86, 88)	(60, 65, 69, 70, 74, 79, 80, 82–85, 87)	
Total cholesterol	(23, 60, 64, 65, 67, 74, 76, 77, 79, 81–84, 86, 88)	(69, 70, 78–80, 85, 87)	
Lipoprotein A	(76, 89)		
Apolipoprotein A		(65, 67)	
Apolipoprotein B	(64, 65, 67)		
**Endothelial function**			
Brachial artery diameter	(90)		
Flow-mediated dilatation	(91–94)	(67, 75, 88, 90)	
**Blood pressure**			
Systolic blood pressure (trials)	(70, 78, 85, 95–97)	(23, 64, 65, 67, 68, 69, 71, 72, 75, 77, 87)	
Diastolic blood pressure (trials)	(71, 78, 96, 97)	(23, 64, 65, 67–70, 72, 75, 77, 85, 87, 95)	
Hypertension (observational)	(37, 38, 73)		
**Body composition and weight**			
Body composition		(98)	
Body weight	(99–105)	(98, 104–108)	
Body mass index (BMI)	(102, 105, 107)	(98, 100, 105, 104, 108)	
Energy intake	(106)		
Fat mass	(104, 100)	(104, 105)	
Overweight/obesity risk	(99, 100)		
Waist circumference	(99, 105)	(58, 98, 100, 104, 107, 108)	
**Hunger and fullness**			
Fullness		(106)	
Hunger	(106)		
Leptin	(109)		
**Glucose and insulin**			
Fasting blood glucose	(58, 102, 110, 111, 112)	(23, 98)	
Glycemic control	(62, 102, 112)		
Insulin sensitivity	(111)	(23)	
Fasting plasma insulin	(111)	(110)	
Adiponectin		(109, 113)	
HOMA-IR	(102, 112)	(98)	
HbA1C		(23, 110–112)	
Glycemic indices	(114)	(98, 114)	
**Inflammation**			
C-reactive protein (CRP)	(115, 116)	(93, 109, 116–118)	
Tumor necrosis factor alpha (TNF-α)	(115, 116)	(93, 109)	
Interleukin 6 and 10 (IL-6, IL-10)	(115)	(93, 109, 116)	
Vascular, intercellular, and endothelial-leukocyte cell adhesion proteins 1 (VCAM-1, ICAM-1, E-selectin)	(115)	(93)	
Antioxidant defense system	(53, 119)		
**Gut microbiota**			
Fecal microbiota		(57, 120)	
**Cognitive function**			
Cognitive performance	(121)	(122)	

Studies are categorized based on biomarker change [favorable/reduced disease risk, neutral (no significant change), or unfavorable/increased risk] and listed as reference numbers.

There is some evidence suggesting a favorable effect of nut consumption on metabolic aspects such as insulin sensitivity (22, 116, 123, 124). Furthermore, since nuts have a high concentration of energy in addition to key nutrients, they have been found to reduce hunger (13, 106), which might be one of the reasons studies have not found nuts to be linked with obesity (99).

## Food-based dietary guidelines and integration

An intake of nuts and seeds of around 20–30 g/day is associated with a range of health benefits including a reduction in the risk of cardiovascular diseases, cancers, and premature deaths as summarized above (21, 22). The consumption is equivalent to approximately a handful of nuts per day. Few studies indicate that increasing intake beyond 30 g is linked with additional benefits. Generally, the health effects seen for different types of nuts and seeds seem to be relatively similar, with parallel findings for many of the health outcomes for almonds, walnuts, hazelnuts, cashews, Brazil nuts, macadamias, and pistachios. Parallel findings are also seen for peanuts and seeds, which have many similarities to tree nuts. Substitution of red and processed meats with nuts is also associated with substantial gains related to cardiovascular disease (21). Nuts and seeds are in many cases consumed as salted and roasted snacks. However, with intake levels of a handful or less, nuts and seeds are rarely among the main contributors to salt in the diet (21). People with allergies to nuts and seeds need to avoid these foods.

For type 2 diabetes and impaired glucose metabolism, the evidence is inconclusive (21, 22). Even if nuts and seeds are highly nutrient- and energy-dense (1), consumption of a serving of nuts and seeds per day is unlikely to contribute to obesity and overweight based on the current evidence (99). Obviously, total energy intake from various foods will be important to weight balance and to prevent overweight and obesity. Further, people with a highly active lifestyle can maintain weight homeostasis with a higher energy intake than people living a sedentary lifestyle (125), and exercise generally has a symbiotic effect on weight balance with a healthy diet (126). Thus, the level of activity is also of importance when balancing intake levels.

Nut consumption might contribute beneficially to cognitive outcomes, prevent cognitive decline, and reduce mortality related to infections and respiratory diseases (12, 42). These potential benefits are highly relevant in an aging population (16).

The current evidence strongly supports nut consumption as part of healthy diets among adults of all ages, particularly to reduce the risk of later harmful health outcomes such as chronic disease. In terms of potential population gains of increased intake of nuts and seeds to at least 20 g/day, it has been estimated that 4.4 million deaths could be averted in North and South America, Europe, Southeast Asia, and the Western Pacific (12). This is estimated from probable reductions in premature deaths related to cardiovascular disease and cancers. Based on consumption and morbidity data in the Nordic and Baltic countries, it is reasonable to assume that many deaths could be averted, as consumption data suggest that intake is often substantially lower than optimal intake levels. A sustained change in the consumption of nuts from none to 25 g/day is associated with an increase in life expectancy of 1–1.5 years for male and female adults in the age range of 40–60 years (127). For children, less evidence is available relating to the effect of nut and seed consumption on disease patterns. Still, the available studies generally show some similarities among children aged 4–18 years to what is presented for adults (128). Intake amounts can be adapted to the age, and the youngest children generally need less energy; however, recommending a handful will give some age adjustment.

Overall, the current evidence supports dietary recommendations to increase nut consumption to a daily serving of 20–30 g nuts and seeds (a handful) for people without allergies to these foods. Different types of nuts seem to contribute positively, and for those without allergies, varying between different nuts might be beneficial as different nuts contain slightly different macro- and micronutrient profiles.
