# Supplementary material for: Nuts and seeds – a scoping review for Nordic Nutrition Recommendations 2023
Source: Food Nutr Res. 2024 Feb 7;68:10.29219/fnr.v68.10483. doi: 10.29219/fnr.v68.10483 (PMC10870978; doi:10.29219/fnr.v68.10483)
Supplement: Supplementary file 1 [file FNR-68-10483-s001.docx]

**Supplementary ­­­Table 1:** Summary of most recent and comprehensive systematic reviews with meta-analysis on nut consumption and associations to diseases and mortality with information of search year, nut type, outcome, number of studies, unit (high versus low [HL] or dose response expressed in gram per day), relative risk ratios, number of participants and cases, heterogeneity, and overall AMSTAR-2 NNR evaluation.

| **First author** | **Search year** | **Ref. no.** | **Nut types** | **Outcome** | **Unit** | **Relative risk ratio (CI)** | **Participants / cases (studies)** | **I^2^** | **AMSTAR-2** |
| --- | --- | --- | --- | --- | --- | --- | --- | --- | --- |
| Afshin | 2013 | 37 | All nuts | CHD | 15g | 0.78 (0.67-0.92) | 141390 / 2101 (4) | 0 | Low |
| Afshin | 2013 | 37 | All nuts | CHD mortality | 15g | 0.76 (0.69-0.84) | 206114 / 6749 (4) | 28 | Low |
| Afshin | 2013 | 37 | All nuts | Stroke | 15g | 0.89 (0.74-1.05) | 155685 / 5544 (4) | 73 | Low |
| Afshin | 2013 | 37 | All nuts | Diabetes mellitus | 15g | 0.87 (0.81-0.94) | 230216 / 13308 (6) | 22 | Low |
| Arnesen | 2022 | 21 | All nuts | CVD | HL | 0.81 (0.75-0.86) | / 61028 (15) | 67 | High |
| Arnesen | 2022 | 21 | All nuts | CVD mortality | HL | 0.77 (0.72-0.82) | / 44792 (15) | 59 | High |
| Arnesen | 2022 | 21 | All nuts | CHD | HL | 0.82 (0.76-0.89) | / 40549 (13) | 64 | High |
| Arnesen | 2022 | 21 | All nuts | CHD mortality | HL | 0.75 (0.65-0.87) | / 8568 (8) | 67 | High |
| Arnesen | 2022 | 21 | All nuts | Stroke | HL | 0.91 (0.85-0.97) | / 22635 (10) | 25 | High |
| Arnesen | 2022 | 21 | All nuts | Ischemic stroke incidence | HL | 0.94 (0.85-1.03) | / 15421 (7) | 37 | High |
| Arnesen | 2022 | 21 | All nuts | Stroke mortality | HL | 0.87 (0.76-1) | / 2566 (6) | 0 | High |
| Arnesen | 2022 | 21 | All nuts | Diabetes mellitus | HL | 0.95 (0.75-1.21) | / 24087 (5) | 82 | High |
| Arnesen | 2022 | 21 | All nuts | CVD | 30g | 0.76 (0.68-0.86) | / 61028 (15) | 67 | High |
| Arnesen | 2022 | 21 | All nuts | CVD mortality | 30g | 0.73 (0.67-0.8) | / 44792 (15) | 59 | High |
| Arnesen | 2022 | 21 | All nuts | CHD | 30g | 0.75 (0.68-0.82) | / 40904 (14) | 64 | High |
| Arnesen | 2022 | 21 | All nuts | CHD mortality | 30g | 0.64 (0.58-0.72) | / 8568 (9) | 67 | High |
| Arnesen | 2022 | 21 | All nuts | Stroke | 30g | 0.93 (0.83-1.04) | / 22635 (11) | 25 | High |
| Arnesen | 2022 | 21 | All nuts | Ischemic stroke incidence | 30g | 0.96 (0.82-1.13) | / 15421 (7) | 37 | High |
| Arnesen | 2022 | 21 | All nuts | Stroke mortality | 30g | 1.1 (0.83-1.45) | / 2566 (6) | 0 | High |
| Aune | 2016 | 12 | All nuts | CVD | HL | 0.81 (0.74-0.89) | 376228 / 18655 (11) | 52 | High |
| Aune | 2016 | 12 | All nuts | CHD | HL | 0.76 (0.69-0.84) | 315397 / 12331 (11) | 48 | High |
| Aune | 2016 | 12 | All nuts | Stroke | HL | 0.89 (0.82-0.97) | 396768 / 9272 (10) | 0 | High |
| Aune | 2016 | 12 | All nuts | Mortality | HL | 0.81 (0.77-0.85) | 819448 / 85870 (15) | 41 | High |
| Aune | 2016 | 12 | All nuts | Cancer mortality | HL | 0.82 (0.74-0.89) | 254240 / 17603 (8) | 28 | High |
| Aune | 2016 | 12 | All nuts | Diabetes mortality | HL | 0.68 (0.52-0.9) | 202751 / 800 (4) | 0 | High |
| Aune | 2016 | 12 | All nuts | Respiratory disease mortality | HL | 0.76 (0.61-0.94) | 130987 / 2551 (3) | 54 | High |
| Aune | 2016 | 12 | All nuts | Infectious disease mortality | HL | 0.79 (0.56-1.11) | 118962 / 397 (2) | 0 | High |
| Aune | 2016 | 12 | All nuts | Kidney disease mortality | HL | 0.69 (0.38-1.25) | 118962 / 367 (2) | 69 | High |
| Aune | 2016 | 12 | All nuts | Neurodegenerative dis. mortality | HL | 0.93 (0.72-1.21) | 130987 / 2056 (3) | 6 | High |
| Aune | 2016 | 12 | Tree nuts | CVD | HL | 0.81 (0.74-0.89) | 130987 / 9456 (3) | 0 | High |
| Aune | 2016 | 12 | Tree nuts | CHD | HL | 0.79 (0.68-0.92) | 130987 / 6394 (3) | 28 | High |
| Aune | 2016 | 12 | Tree nuts | Stroke | HL | 0.89 (0.82-0.97) | 130987 / 2130 (3) | 0 | High |
| Aune | 2016 | 12 | Tree nuts | Mortality | HL | 0.8 (0.74-0.86) | 202751 / 42508 (4) | 58 | High |
| Aune | 2016 | 12 | Tree nuts | Cancer mortality | HL | 0.82 (0.76-0.9) | 130987 / 14210 (3) | 0 | High |
| Aune | 2016 | 12 | Tree nuts | Diabetes mortality | HL | 1.19 (0.74-1.89) | 130987 / 462 (3) | 0 | High |
| Aune | 2016 | 12 | Tree nuts | Respiratory disease mortality | HL | 0.89 (0.74-1.07) | 130987 / 2551 (3) | 0 | High |
| Aune | 2016 | 12 | Tree nuts | Infectious disease mortality | HL | 0.73 (0.47-1.13) | 118962 / 397 (2) | 0 | High |
| Aune | 2016 | 12 | Tree nuts | Kidney disease mortality | HL | 0.65 (0.4-1.03) | 118962 / 367 (2) | 0 | High |
| Aune | 2016 | 12 | Tree nuts | Neurodegenerative dis. mortality | HL | 0.94 (0.75-1.18) | 130987 / 2056 (3) | 14 | High |
| Aune | 2016 | 12 | Peanuts | CVD | HL | 0.81 (0.75-0.87) | 265252 / 12043 (5) | 15 | High |
| Aune | 2016 | 12 | Peanuts | CHD | HL | 0.76 (0.69-0.82) | 265252 / 7025 (5) | 0 | High |
| Aune | 2016 | 12 | Peanuts | Stroke | HL | 0.83 (0.69-1) | 265252 / 3315 (5) | 46 | High |
| Aune | 2016 | 12 | Peanuts | Mortality | HL | 0.85 (0.82-0.89) | 265252 / 44396 (5) | 18 | High |
| Aune | 2016 | 12 | Peanuts | Cancer mortality | HL | 0.93 (0.87-0.99) | 265252 / 17742 (5) | 19 | High |
| Aune | 2016 | 12 | Peanuts | Diabetes mortality | HL | 0.84 (0.6-1.19) | 202751 / 800 (4) | 43 | High |
| Aune | 2016 | 12 | Peanuts | Respiratory disease mortality | HL | 0.77 (0.63-0.93) | 130987 / 2551 (3) | 39 | High |
| Aune | 2016 | 12 | Peanuts | Infectious disease mortality | HL | 1.01 (0.83-1.23) | 118962 / 397 (2) | 13 | High |
| Aune | 2016 | 12 | Peanuts | Kidney disease mortality | HL | 0.52 (0.27-0.97) | 118962 / 367 (2) | 0 | High |
| Aune | 2016 | 12 | Peanuts | Neurodegenerative dis. mortality | HL | 0.94 (0.72-1.23) | 130987 / 2056 (3) | 46 | High |
| Aune | 2016 | 12 | All nuts | CVD | 28g | 0.79 (0.7-0.88) | 376228 / 18655 (12) | 60 | High |
| Aune | 2016 | 12 | All nuts | CHD | 28g | 0.71 (0.63-0.8) | 315397 / 12331 (11) | 47 | High |
| Aune | 2016 | 12 | All nuts | Stroke | 28g | 0.93 (0.83-1.05) | 396768 / 9272 (11) | 14 | High |
| Aune | 2016 | 12 | All nuts | Mortality | 28g | 0.78 (0.72-0.84) | 819448 / 85870 (16) | 66 | High |
| Aune | 2016 | 12 | All nuts | Cancer mortality | 28g | 0.85 (0.76-0.94) | 254240 / 17603 (8) | 42 | High |
| Aune | 2016 | 12 | All nuts | Diabetes mortality | 28g | 0.61 (0.43-0.88) | 202751 / 800 (4) | 0 | High |
| Aune | 2016 | 12 | All nuts | Respiratory disease mortality | 28g | 0.48 (0.26-0.89) | 130987 / 2551 (3) | 61 | High |
| Aune | 2016 | 12 | All nuts | Infectious disease mortality | 28g | 0.25 (0.07-0.85) | 118962 / 397 (2) | 54 | High |
| Aune | 2016 | 12 | All nuts | Kidney disease mortality | 28g | 0.27 (0.04-1.91) | 118962 / 367 (2) | 61 | High |
| Aune | 2016 | 12 | All nuts | Neurodegenerative dis. mortality | 28g | 0.65 (0.4-1.08) | 130987 / 2056 (3) | 6 | High |
| Aune | 2016 | 12 | Tree nuts | CVD | 28g | 0.75 (0.67-0.84) | 130987 / 9456 (3) | 0 | High |
| Aune | 2016 | 12 | Tree nuts | CHD | 28g | 0.73 (0.63-0.85) | 130987 / 6394 (3) | 0 | High |
| Aune | 2016 | 12 | Tree nuts | Stroke | 28g | 0.89 (0.69-1.14) | 130987 / 2130 (3) | 0 | High |
| Aune | 2016 | 12 | Tree nuts | Mortality | 28g | 0.82 (0.75-0.9) | 202751 / 42508 (4) | 70 | High |
| Aune | 2016 | 12 | Tree nuts | Cancer mortality | 28g | 0.8 (0.72-0.89) | 130987 / 14210 (3) | 0 | High |
| Aune | 2016 | 12 | Tree nuts | Diabetes mortality | 28g | 1.23 (0.68-2.25) | 130987 / 462 (3) | 0 | High |
| Aune | 2016 | 12 | Tree nuts | Respiratory disease mortality | 28g | 0.79 (0.62-1.01) | 130987 / 2551 (3) | 0 | High |
| Aune | 2016 | 12 | Tree nuts | Infectious disease mortality | 28g | 0.64 (0.36-1.13) | 118962 / 397 (2) | 0 | High |
| Aune | 2016 | 12 | Tree nuts | Kidney disease mortality | 28g | 0.66 (0.36-1.22) | 118962 / 367 (2) | 0 | High |
| Aune | 2016 | 12 | Tree nuts | Neurodegenerative dis. mortality | 28g | 0.81 (0.58-1.12) | 130987 / 2056 (3) | 26 | High |
| Aune | 2016 | 12 | Peanuts | CVD | 28g | 0.64 (0.5-0.81) | 265252 / 12043 (5) | 77 | High |
| Aune | 2016 | 12 | Peanuts | CHD | 28g | 0.69 (0.57-0.84) | 265252 / 7025 (5) | 45 | High |
| Aune | 2016 | 12 | Peanuts | Stroke | 28g | 0.63 (0.41-0.95) | 265252 / 3315 (5) | 78 | High |
| Aune | 2016 | 12 | Peanuts | Mortality | 28g | 0.77 (0.69-0.86) | 265252 / 44396 (5) | 64 | High |
| Aune | 2016 | 12 | Peanuts | Cancer mortality | 28g | 0.92 (0.82-1.03) | 265252 / 17742 (5) | 30 | High |
| Aune | 2016 | 12 | Peanuts | Diabetes mortality | 28g | 0.73 (0.45-1.2) | 202751 / 800 (4) | 15 | High |
| Aune | 2016 | 12 | Peanuts | Respiratory disease mortality | 28g | 0.69 (0.53-0.91) | 130987 / 2551 (3) | 50 | High |
| Aune | 2016 | 12 | Peanuts | Infectious disease mortality | 28g | 1.03 (0.81-1.31) | 118962 / 397 (2) | 3 | High |
| Aune | 2016 | 12 | Peanuts | Kidney disease mortality | 28g | 0.42 (0.24-0.73) | 118962 / 367 (2) | 0 | High |
| Aune | 2016 | 12 | Peanuts | Neurodegenerative dis. mortality | 28g | 0.92 (0.65-1.31) | 130987 / 2056 (3) | 49 | High |
| Becerra-Tomás | 2019 | 38 | All nuts | CVD | HL | 0.85 (0.8-0.91) | 210836 / 14136 (3) | 0 | High |
| Becerra-Tomás | 2019 | 38 | All nuts | CVD mortality | HL | 0.77 (0.72-0.82) | 413727 / 14475 (14) | 3 | High |
| Becerra-Tomás | 2019 | 38 | All nuts | CHD | HL | 0.82 (0.69-0.96) | 275812 / 12654 (7) | 74 | High |
| Becerra-Tomás | 2019 | 38 | All nuts | CHD mortality | HL | 0.76 (0.67-0.86) | 396041 / 7877 (12) | 46 | High |
| Becerra-Tomás | 2019 | 38 | All nuts | Stroke | HL | 1 (0.92-1.09) | 302888 / 12646 (7) | 0 | High |
| Becerra-Tomás | 2019 | 38 | All nuts | Stroke mortality | HL | 0.83 (0.75-0.93) | 351618 / 2332 (11) | 0 | High |
| Becerra-Tomás | 2019 | 38 | All nuts | Hemorrhagic stroke incidence | HL | 1.02 (0.77-1.34) | 188750 / 3088 (5) | 15 | High |
| Becerra-Tomás | 2019 | 38 | All nuts | Ischemic stroke incidence | HL | 0.99 (0.89-1.1) | 302423 / 8401 (7) | 0 | High |
| Becerra-Tomás | 2019 | 38 | All nuts | Atrial fibrillation | HL | 0.85 (0.73-0.99) | 53965 / 10867 (2) | 0 | High |
| Becerra-Tomás | 2019 | 38 | All nuts | Heart failure | HL | 1 (0.86-1.16) | 53887 / 4253 (2) | 0 | High |
| Becerra-Tomás | 2019 | 38 | All nuts | CVD | 28g | 0.87 (0.81-0.93) | 210836 / 14136 (3) | 0 | High |
| Becerra-Tomás | 2019 | 38 | All nuts | CVD mortality | 28g | 0.71 (0.61-0.84) | 413727 / 14475 (14) | 3 | High |
| Becerra-Tomás | 2019 | 38 | All nuts | CHD | 28g | 0.75 (0.64-0.88) | 275812 / 12654 (7) | 74 | High |
| Becerra-Tomás | 2019 | 38 | All nuts | CHD mortality | 28g | 0.67 (0.52-0.87) | 396041 / 7877 (12) | 46 | High |
| Becerra-Tomás | 2019 | 38 | All nuts | Stroke | 28g | 1.06 (0.97-1.15) | 302888 / 12646 (7) | 0 | High |
| Becerra-Tomás | 2019 | 38 | All nuts | Stroke mortality | 28g | 1.01 (0.88-1.18) | 351618 / 2332 (11) | 0 | High |
| Becerra-Tomás | 2019 | 38 | All nuts | Hemorrhagic stroke incidence | 28g | 1.05 (0.77-1.43) | 188750 / 3088 (5) | 15 | High |
| Becerra-Tomás | 2019 | 38 | All nuts | Ischemic stroke incidence | 28g | 1.06 (0.86-1.31) | 302423 / 8401 (7) | 0 | High |
| Becerra-Tomás | 2019 | 38 | Tree nuts | CVD | HL | 0.85 (0.79-0.91) | 210836 / 14136 (3) | 0 | High |
| Becerra-Tomás | 2019 | 38 | Tree nuts | CHD | HL | 0.77 (0.7-0.84) | 210836 / 8390 (3) | 61 | High |
| Becerra-Tomás | 2019 | 38 | Tree nuts | Stroke | HL | 1 (0.89-1.11) | 210836 / 5910 (3) | 0 | High |
| Becerra-Tomás | 2019 | 38 | Tree nuts | Stroke mortality | HL | 0.93 (0.77-1.13) | 118962 / 1851 (3) | 0 | High |
| Becerra-Tomás | 2019 | 38 | Peanuts | CVD | HL | 0.87 (0.81-0.93) | 210836 / 14136 (3) | 0 | High |
| Becerra-Tomás | 2019 | 38 | Peanuts | CVD mortality | HL | 0.77 (0.7-0.85) | 134265 / 5572 (2) | 0 | High |
| Becerra-Tomás | 2019 | 38 | Peanuts | CHD | HL | 0.85 (0.79-0.92) | 210836 / 8390 (3) | 0 | High |
| Becerra-Tomás | 2019 | 38 | Peanuts | CHD mortality | HL | 0.75 (0.64-0.88) | 134265 / 2119 (2) | 0 | High |
| Becerra-Tomás | 2019 | 38 | Peanuts | Stroke | HL | 0.9 (0.81-0.99) | 210836 / 5910 (3) | 13 | High |
| Becerra-Tomás | 2019 | 38 | Peanuts | Stroke mortality | HL | 0.83 (0.73-0.95) | 253227 / 3036 (4) | 57 | High |
| Becerra-Tomás | 2019 | 38 | Walnuts | CVD | HL | 0.81 (0.71-0.92) | 144021 / 5255 (3) | 3 | High |
| Becerra-Tomás | 2019 | 38 | Walnuts | CHD | HL | 0.79 (0.66-0.94) | 144021 / 2685 (3) | 4 | High |
| Becerra-Tomás | 2019 | 38 | Walnuts | Stroke | HL | 0.85 (0.71-1.02) | 144021 / 5910 (3) | 19 | High |
| Becerra-Tomás | 2021 | 15 | All nuts | Diabetes mellitus | HL | 1.04 (0.94-1.15) | 194168 / (4) | 60 | High |
| Becerra-Tomás | 2021 | 15 | Tree nuts | Diabetes mellitus | HL | 0.98 (0.87-1.11) | 137956 / (2) | 0 | High |
| Becerra-Tomás | 2021 | 15 | Walnuts | Diabetes mellitus | HL | 0.76 (0.62-0.94) | 137956 / (1) | 0 | High |
| Becerra-Tomás | 2021 | 15 | Peanuts | Diabetes mellitus | HL | 0.95 (0.87-1.04) | 202147 / (3) | 72 | High |
| Bechthold | 2017 | 39 | All nuts | CHD | HL | 0.8 (0.62-1.03) | / 5480 (4) | 79 | High |
| Bechthold | 2017 | 39 | All nuts | Stroke | HL | 0.94 (0.85-1.05) | / 7490 (6) | 18 | High |
| Bechthold | 2017 | 39 | All nuts | Heart failure | HL | 0.99 (0.86-1.15) | / 3613 (3) | 57 | High |
| Bechthold | 2017 | 39 | All nuts | CHD | 28g | 0.67 (0.43-1.05) | / 5480 (4) | 85 | High |
| Bechthold | 2017 | 39 | All nuts | Stroke | 28g | 0.99 (0.84-1.17) | / 7490 (6) | 45 | High |
| Bechthold | 2017 | 39 | All nuts | Heart failure | 28g | 1.09 (0.97-1.22) | / 3613 (2) | 0 | High |
| Brandt (van den) | 2014 | 35 | All nuts | Cancer mortality | HL | 0.85 (0.77-0.93) | 247030 / 14340 (4) | 17 | Critically |
| Brandt (van den) | 2014 | 35 | All nuts | Respiratory disease mortality | HL | 0.71 (0.58-0.86) | 239814 / 2551 (3) | 5 | Critically |
| Chen | 2016 | 11 | All nuts | CVD mortality | HL | 0.75 (0.71-0.79) | 524610 / 19574 (16) | 0 | Low |
| Chen | 2016 | 11 | All nuts | CHD mortality | HL | 0.73 (0.67-0.8) | 429833 / 10083 (13) | 14 | Low |
| Chen | 2016 | 11 | All nuts | Stroke mortality | HL | 0.82 (0.73-0.91) | 449293 / 4398 (12) | 0 | Low |
| Chen | 2016 | 11 | All nuts | Mortality | HL | 0.81 (0.78-0.84) | 498730 / 66568 (16) | 22 | Low |
| Chen | 2016 | 11 | All nuts | Cancer mortality | HL | 0.87 (0.8-0.93) | 451589 / 21353 (11) | 26 | Low |
| Chen | 2016 | 11 | Tree nuts | CVD mortality | HL | 0.81 (0.74-0.89) | 130987 / 9456 (3) | 0 | Low |
| Chen | 2016 | 11 | Tree nuts | CHD mortality | HL | 0.79 (0.68-0.92) | 130987 / 6394 (3) | 28 | Low |
| Chen | 2016 | 11 | Tree nuts | Stroke mortality | HL | 0.93 (0.77-1.13) | 130987 / 2130 (3) | 0 | Low |
| Chen | 2016 | 11 | Tree nuts | Mortality | HL | 0.83 (0.77-0.89) | 130987 / 36252 (3) | 0 | Low |
| Chen | 2016 | 11 | Tree nuts | Cancer mortality | HL | 0.82 (0.76-0.9) | 130987 / 14210 (3) | 0 | Low |
| Chen | 2016 | 11 | Peanuts | CVD mortality | HL | 0.78 (0.73-0.85) | 265252 / 12052 (5) | 18 | Low |
| Chen | 2016 | 11 | Peanuts | CHD mortality | HL | 0.76 (0.69-0.82) | 265252 / 7025 (5) | 0 | Low |
| Chen | 2016 | 11 | Peanuts | Stroke mortality | HL | 0.83 (0.71-0.97) | 265252 / 3315 (5) | 46 | Low |
| Chen | 2016 | 11 | Peanuts | Mortality | HL | 0.85 (0.81-0.89) | 265252 / 44396 (5) | 34 | Low |
| Chen | 2016 | 11 | Peanuts | Cancer mortality | HL | 0.93 (0.87-0.99) | 265252 / 17742 (5) | 19 | Low |
| Chen | 2016 | 11 | All nuts | CVD mortality | 28g/week | 0.94 (0.93-0.96) | 509871 / 20362 (16) | 60 | Low |
| Chen | 2016 | 11 | All nuts | CHD mortality | 28g/week | 0.94 (0.93-0.96) | 412892 / 10399 (13) | 34 | Low |
| Chen | 2016 | 11 | All nuts | Stroke mortality | 28g/week | 0.95 (0.91-1) | 432352 / 4831 (12) | 61 | Low |
| Chen | 2016 | 11 | All nuts | Mortality | 28g/week | 0.96 (0.94-0.97) | 766470 / 81034 (18) | 72 | Low |
| Chen | 2016 | 11 | All nuts | Cancer mortality | 28g/week | 0.97 (0.96-0.99) | 434405 / 21302 (10) | 23 | Low |
| Chen | 2016 | 11 | Tree nuts | CVD mortality | 28g/week | 0.91 (0.86-0.95) | 130987 / 9456 (3) | 0 | Low |
| Chen | 2016 | 11 | Tree nuts | CHD mortality | 28g/week | 0.89 (0.84-0.95) | 130987 / 6394 (3) | 15 | Low |
| Chen | 2016 | 11 | Tree nuts | Stroke mortality | 28g/week | 0.96 (0.87-1.06) | 130987 / 2130 (3) | 0 | Low |
| Chen | 2016 | 11 | Tree nuts | Mortality | 28g/week | 0.91 (0.89-0.94) | 130987 / 36252 (3) | 0 | Low |
| Chen | 2016 | 11 | Tree nuts | Cancer mortality | 28g/week | 0.92 (0.88-0.96) | 130987 / 14210 (3) | 0 | Low |
| Chen | 2016 | 11 | Peanuts | CVD mortality | 28g/week | 0.84 (0.77-0.93) | 265252 / 12052 (5) | 80 | Low |
| Chen | 2016 | 11 | Peanuts | CHD mortality | 28g/week | 0.87 (0.81-0.95) | 265252 / 7025 (5) | 53 | Low |
| Chen | 2016 | 11 | Peanuts | Stroke mortality | 28g/week | 0.84 (0.73-0.96) | 265252 / 3315 (5) | 72 | Low |
| Chen | 2016 | 11 | Peanuts | Mortality | 28g/week | 0.91 (0.87-0.95) | 265252 / 44396 (5) | 64 | Low |
| Chen | 2016 | 11 | Peanuts | Cancer mortality | 28g/week | 0.97 (0.93-1.01) | 265252 / 17742 (5) | 23 | Low |
| Grosso | 2014 | 14 | All nuts | CVD mortality | HL | 0.71 (0.62-0.81) | 354933 / 7775 (9) | 25 | Critically |
| Grosso | 2014 | 14 | All nuts | Mortality | HL | 0.77 (0.69-0.87) | 354933 / 44636 (9) | 56 | Critically |
| Grosso | 2014 | 14 | All nuts | Cancer mortality | HL | 0.86 (0.75-0.98) | 354933 / 10423 (9) | 32 | Critically |
| Grosso | 2014 | 14 | All nuts | CVD mortality | 28g | 0.61 (0.42-0.91) | 354933 / 7775 (9) | 74 | Critically |
| Grosso | 2014 | 14 | All nuts | Mortality | 28g | 0.73 (0.6-0.88) | 354933 / 44636 (9) | 53 | Critically |
| Grosso | 2014 | 14 | All nuts | Cancer mortality | 28g | 0.69 (0.33-1.45) | 354933 / 10423 (9) | 70 | Critically |
| Guo | 2013 | 38 | All nuts | Diabetes mellitus | HL | 0.98 (0.84-1.15) | 263663 / 11 580 (6) | 68 | Low |
| Guo | 2013 | 38 | All nuts | Diabetes mellitus | 28g | 1.04 (0.95-1.14) | 263663 / 11 580 (6) | 58 | Low |
| Li | 2017 | 73 | All nuts | Metabolic syndrome | 28g/week | 0.96 (0.92-0.99) | 20666 / 4625 (6) | 0 | Low |
| Li | 2017 | 73 | All nuts | Overweight or obesity- | 28g/week | 0.97 (0.95-0.98) | 203591 / 28678 (6) | 0 | Low |
| Li | 2017 | 73 | All nuts | Obesity | 28g/week | 0.95 (0.89-1.02) | 178633 / 18557 (6) | 74 | Low |
| Luo | 2013 | 39 | All nuts | CVD | 28g | 0.71 (0.59-0.85) | / 8862 (4) | 49 | High |
| Luo | 2013 | 39 | All nuts | CHD | 28g | 0.72 (0.64-0.81) | / 6623 (6) | 0 | High |
| Luo | 2013 | 39 | All nuts | Stroke | 28g | 0.91 (0.81-1.02) | / 6487 (5) | 20 | High |
| Luo | 2013 | 39 | All nuts | Mortality | 28g | 0.83 (0.76-0.91) | / 48818 (11) | 62 | High |
| Luo | 2013 | 39 | All nuts | Diabetes mellitus | 28g | 0.88 (0.84-0.92) | / 12655 (8) | 68 | High |
| Ma | 2014 | 44 | All nuts | CHD | HL | 0.66 (0.58-0.75) | 347477 / 6127 (13) | 40 | Low |
| Ma | 2014 | 44 | All nuts | CHD | 28g | 0.7 (0.58-0.83) | / 4886 (7) | 0 | Low |
| Mayhew | 2015 | 45 | All nuts | CVD mortality | HL | 0.73 (0.68-0.78) | 243795 / 13726 (5) | 16 | Low |
| Mayhew | 2015 | 45 | All nuts | CHD | HL | 0.66 (0.48-0.91) | 123971 / 4757 (3) | 88 | Low |
| Mayhew | 2015 | 45 | All nuts | Stroke | HL | 1.05 (0.69-1.61) | 157826 / 4318 (2) | 77 | Low |
| Mayhew | 2015 | 45 | All nuts | Stroke mortality | HL | 0.83 (0.69-1) | 159322 / 2166 (3) | 0 | Low |
| Mayhew | 2015 | 45 | All nuts | Mortality | HL | 0.81 (0.77-0.85) | 277432 / 49232 (10) | 43 | Low |
| Mayhew | 2015 | 45 | All nuts | CVD | 16g | 0.72 (0.55-0.96) | 6309 / 634 (1) | 0 | Low |
| Mayhew | 2015 | 45 | All nuts | CVD mortality | 16g | 0.78 (0.63-1) | 243795 / 13726 (5) | 16 | Low |
| Mayhew | 2015 | 45 | All nuts | CHD mortality | 16g | 0.78 (0.57-1.08) | 278584 / 8454 (7) | 0 | Low |
| Mayhew | 2015 | 45 | All nuts | Stroke mortality | 16g | 0.85 (0.55-1.31) | 159322 / 2166 (3) | 0 | Low |
| Mayhew | 2015 | 45 | All nuts | Mortality | 16g | 0.81 (0.75-0.92) | 277432 / 49232 (10) | 43 | Low |
| Naghshi | 2020 | 33 | All nuts | Cancer mortality | HL | 0.87 (0.82-0.91) | 819851 / 48038 (10) | 23 | High |
| Naghshi | 2020 | 33 | Tree nuts | Cancer mortality | HL | 0.82 (0.76-0.9) | / (3) | 0 | High |
| Naghshi | 2020 | 33 | Peanuts | Cancer mortality | HL | 0.92 (0.86-0.99) | / (4) | 34 | High |
| Naghshi | 2020 | 33 | All nuts | Cancer mortality | 5g | 0.96 (0.95-0.98) | / (10) | 30 | High |
| Naghshi | 2020 | 33 | Peanuts | Cancer mortality | 5g | 0.97 (0.92-1.02) | / (4) | 49 | High |
| Schwingshackl | 2016 | 46 | All nuts | Mortality | HL | 0.8 (0.74-0.86) | / 80204 (16) | 84 | High |
| Schwingshackl | 2016 | 46 | All nuts | Mortality | 28g | 0.76 (0.69-0.84) | / 80204 (16) | 82 | High |
| Schwingshackl | 2017 | 36 | All nuts | Diabetes mellitus | HL | 0.95 (0.85-1.05) | / 27016 (8) | 67 | High |
| Schwingshackl | 2017 | 36 | All nuts | Diabetes mellitus | 28g | 0.89 (0.71-1.12) | / 27016 (8) | 77 | High |
| Shao | 2016 | 48 | All nuts | Stroke | HL | 0.88 (0.8-0.97) | / (14) | 0 | High |
| Shao | 2016 | 48 | All nuts | Stroke mortality | HL | 0.81 (0.72-0.91) | / () | 0 | High |
| Shao | 2016 | 48 | All nuts | Stroke | 12g | 0.86 (0.79-0.94) | / (11) | 0 | High |
| Shi | 2014 | 49 | All nuts | Stroke | HL | 0.9 (0.81-0.99) | 228799 / 5669 (8) | 0 | Low |
| Shi | 2014 | 49 | All nuts | Hemorrhagic stroke incidence | HL | 1.17 (0.54-2.54) | 210472 / 5601 (3) | 78 | Low |
| Shi | 2014 | 49 | All nuts | Ischemic stroke incidence | HL | 0.97 (0.84-1.1) | 210472 / 5601 (3) | 0 | Low |
| Weng | 2014 | 50 | All nuts | CHD | HL | 0.68 (0.59-0.78) | / 6302 (14) | 63 | Low |
| Weng | 2014 | 50 | All nuts | CHD | 28g/week | 0.9 (0.87-0.94) | / 6127 (13) | 68 | Low |
| Wu | 2013 | 31 | All nuts | Diabetes mellitus | HL | 0.98 (0.84-1.14) | / (5) | 74 | Critically |
| Zhang | 2018 | 32 | All nuts | Metabolic syndrome | HL | 0.84 (0.76-0.92) | 89224 / (11) | 79 | Low |
| Zhang | 2018 | 32 | Tree nuts | Metabolic syndrome | HL | 0.97 (0.94-1) | / (4) | 0 | Low |
| Zhang | 2018 | 32 | Peanuts | Metabolic syndrome | HL | 1.01 (0.96-1.06) | / (2) | 0 | Low |
| Zhang | 2019 | 35 | All nuts | Cancer mortality | HL | 0.9 (0.88-0.92) | / 49161 (9) | 14 | High |
| Zhang | 2014 | 52 | All nuts | Stroke | HL | 0.9 (0.83-0.98) | 476181  / (9) | 0 | Low |
| Zhang | 2014 | 52 | All nuts | Hemorrhagic stroke incidence | HL | 1.53 (0.97-2.41) | 194307 / 5454 (2) | 0 | Low |
| Zhang | 2014 | 52 | All nuts | Ischemic stroke incidence | HL | 0.9 (0.74-1.09) | 210472 / 5601 (3) | 0 | Low |

* Abbreviations: CHD: coronary heart disease; CVD: cardiovascular disease; 28g: 28 grams/day, 28g/w: 28 grams/week;
